# Test-retest analysis of cerebral oxygen extraction estimates in healthy volunteers: comparison of methods based on quantitative susceptibility mapping and dynamic susceptibility contrast magnetic resonance imaging

**DOI:** 10.1016/j.heliyon.2022.e12364

**Published:** 2022-12-16

**Authors:** Ronnie Wirestam, Anna Lundberg, Arthur Chakwizira, Danielle van Westen, Linda Knutsson, Emelie Lind

**Affiliations:** a Department of Medical Radiation Physics, Lund University, Lund, Sweden; b Department of Diagnostic Radiology, Lund University, Lund, Sweden; c Image and Function, Skåne University Hospital, Lund, Sweden; d F.M. Kirby Research Center for Functional Brain Imaging, Kennedy Krieger Institute, Baltimore, MD, United States; e Russell H. Morgan Department of Radiology and Radiological Science, Johns Hopkins University School of Medicine, Baltimore, MD, United States

**Keywords:** Magnetic resonance imaging, Contrast media, Cerebrovascular circulation, Hemodynamics, Oxygen consumption, Magnetometry

## Abstract

**Background:**

Estimation of the oxygen extraction fraction (OEF) by quantitative susceptibility mapping (QSM) magnetic resonance imaging (MRI) is promising but requires systematic evaluation. Extraction of OEF-related information from the tissue residue function in dynamic susceptibility contrast MRI (DSC-MRI) has also been proposed. In this study, whole-brain OEF repeatability was investigated, as well as the relationships between QSM-based OEF and DSC-MRI-based parameters, i.e., mean transit time (MTT) and an oxygen extraction index, referred to as apparent OEF (AOEF).

**Method:**

Test-retest data were obtained from 20 healthy volunteers at 3 T. QSM maps were reconstructed from 3D gradient-echo MRI phase data, using morphology-enabled dipole inversion. DSC-MRI was accomplished using gradient-echo MRI at a temporal resolution of 1.24 s.

**Results:**

The whole-brain QSM-based OEF was (40.4±4.8) % and, in combination with a previously published cerebral blood flow (CBF) estimate, this corresponds to a cerebral metabolic rate of oxygen level of CMRO_2_ = 3.36 ml O_2_/min/100 g. The intra-class correlation coefficient [ICC(2,1)] for OEF test-retest data was 0.73. The MTT-versus-OEF and AOEF-versus-OEF relationships showed correlation coefficients of 0.61 (p = 0.004) and 0.52 (p = 0.019), respectively.

**Discussion:**

QSM-based OEF showed a convincing absolute level and good test-retest results in terms of the ICC. Moderate to good correlations between QSM-based OEF and DSC-MRI-based parameters were observed. The present results constitute an indicator of the level of robustness that can be achieved without applying extraordinary resources in terms of MRI equipment, imaging protocol, QSM reconstruction, and OEF analysis.

## 1 Introduction

Magnetic resonance imaging (MRI) methods for estimation of cerebral blood flow (CBF) as well as the venous oxygen saturation level, closely related to the oxygen extraction fraction (OEF), are well established, offering the opportunity to assess also the cerebral metabolic rate of oxygen (CMRO_2_). MRI susceptometry, based on phase information, is a common approach for quantification of the venous oxygen saturation levels [1, 2], and a more recent expansion of this technique is based on quantitative susceptibility mapping (QSM) [3, 4, 5]. For example, global or whole brain OEF estimation using QSM has recently received substantial attention in the characterization of multiple sclerosis (MS) lesions [6], and promising attempts to achieve regional evaluation or visualization have also been introduced [7, 8]. The use of QSM for OEF estimation is clearly well documented, but QSM reconstruction is a delicate mathematical procedure known to be sensitive to noise, choice of algorithm, settings of regularization parameters, choice of brain mask, etc., and systematic evaluation of acquired OEF estimates is thus warranted.

With regard to MRI-based CBF assessment, the most common method in clinical environments is dynamic susceptibility contrast MRI (DSC-MRI) [9]. DSC-MRI is generally a robust and time efficient technique, but a number of methodological impediments that hamper CBF quantification in absolute terms need to be considered [10]. DSC-MRI data processing includes retrieval of the tissue residue function, by deconvolution of the tissue contrast agent (CA) concentration curve with the arterial input function (AIF). This implies that DSC-MRI is, in principle, able to provide information related to the blood transit time distribution and, hypothetically, also oxygen extraction occurring during the passage from the arterial inlet to the venous outlet of the local capillary network. An intriguing theoretical framework for assessment of an OEF-related metric (referred to as oxygen extraction capacity), based on DSC-MRI capillary transit time distributions, has been proposed by Jespersen & Østergaard and Mouridsen et al. [11, 12].

However, a crucial prerequisite for accurate estimation of the shape of the tissue residue function is a reliable deconvolution algorithm. The true residue function is, by definition, characterized by non-negative values and strict decrease, while the most commonly used deconvolution algorithms, based on singular value decomposition [9, 13], tend to return residue function estimates that suffer from severe oscillations [10]. Hence, improved deconvolution algorithms, with the ability to return physiologically plausible residue functions in DSC-MRI, have attracted recent attention [e.g. 14, 15, 16]. The use of a control point interpolation (CPI) method, as introduced by Mehndiratta et al. [15], is an attractive approach to impose the desired constraints on the output, while at the same time avoiding the need for a parametric or model-dependent algorithm. However, in CPI, the residue function must pass through all control points. Hence, as a further development, deconvolution based on Bézier curves was recently introduced [16]. The main advantage with such an algorithm is that a Bézier curve needs only be bounded by the convex hull of its control points, which significantly reduces the number of optimization parameters.

The tissue residue function or transit time distribution, obtained by DSC-MRI, is not expected to carry all pieces of information that are required to quantify true OEF, but the shape of the tissue residue function may still provide complementary understanding of tissue oxygenation and viability. Hence, attempts to compare such an OEF-related metric with an established OEF method are indeed of interest. In the present study, both conventional mean transit time (MTT) and an OEF-related metric, below referred to as apparent OEF (AOEF), retrieved from DSC-MRI data, were directly compared with the corresponding QSM-based whole-brain OEF estimates in a group of healthy volunteers. Additionally, test-retest analyses of the extracted parameters were carried out.

## 2 Material and methods

### 2.1 Theory

#### 2.1.1 QSM-based OEF estimation

The underlying susceptibility distribution χ(r) of the tissue is related to a corresponding phase shift Δ∅(r) according to Eq. (1) [17]:

(1) $$\Delta \emptyset \left(r\right)=\gamma \cdot B_{0}\cdot TE\cdot d⊗\chi \left(r\right),$$

where r is the voxel position given in spherical coordinates, γ is the gyromagnetic ratio, $B_{0}$ is the magnetic flux density of the main magnetic field, TE is the echo time of a gradient-echo (GRE) sequence, $d=\frac{3\;\cos^{2}\;\vartheta -1}{4\pi r^{3}}$ is the z-component of the unit dipole kernel (ϑ is the observation angle relative the main magnetic field) and the symbol “⊗” denotes convolution. The mathematical inversion required to obtain the spatial susceptibility distribution from measured phase is referred to as QSM [18, 19]. Assuming that the arterial oxygen saturation is 100%, OEF can be expressed as OEF=1−Y, where Y is the fractional venous oxygen saturation level. Eq. (2) shows the relationship between OEF and the susceptibility difference Δχ between the surrounding tissue (here assumed to represent arterial blood) and the venous vessel [1]:

(2) $$OEF=\frac{\Delta \chi }{\Delta \chi_{do}\cdot Hct},$$

where $\Delta \chi_{do}=0.193\cdot 4\pi$ ppm (in SI units) is the susceptibility difference per unit hematocrit between fully deoxygenated and fully oxygenated blood [20]. Hct is the fractional hematocrit, in this study assumed to be 0.42 for males and 0.40 for females [21].

The cerebral metabolic rate of oxygen is given by:

(3) $${CMRO}_{2}=OEF\cdot CBF\cdot C_{a}=OEF\cdot CBF\cdot MCHC\cdot Hct\cdot c$$

C_a_ = [Hb]⋅c = MCHC⋅Hct⋅c is the oxygen concentration of blood, where [Hb] is the hemoglobin concentration in the blood, *c* is the Hb-carrying capacity of oxygen and MCHC is the mean corpuscular Hb concentration in red blood cells. In the calculations presented here, MCHC = 34 g/dl [21] and c = 1.368 ml/g [22] were used.

#### 2.1.2 Dynamic susceptibility contrast MRI

DSC-MRI [9, 10, 23] is based on monitoring of the CA bolus passage in tissue and artery, after intravenous CA injection in a peripheral vein. Measured contrast agent concentration is given by C_m_(t) = -ln[S(t)/S_0_]/(TE r2∗), where r2∗ is the T2∗ relaxivity, t is time and S_0_ is the baseline signal. Gadolinium-based CAs are plasma tracers, so a factor k_H_ is applied, accounting for brain density and hematocrit in large and small blood vessels, i.e., tissue concentration is C_t_(t) = k_H_⋅C_m_(t). The general tracer-kinetic relationship in Eq. (4) is employed for quantification.

(4) $$C_{t}\left(t\right)=CBF\left[R\left(t\right)⊗C_{art}\left(t\right)\right]\text{,}$$

where C_t_(t) is the tissue CA concentration and C_art_(t) is the arterial input function (AIF). CBF⋅R(t), obtained by deconvolution, is the tissue impulse response function and R(t) is the tissue residue function. The transit time distribution is given by h(t) = -dR(t)/dt and the MTT was obtained according to Eq. (5):

(5) $$MTT=\int_{0}^{\infty }R\left(t\right)dt$$

An OEF-related DSC-MRI metric can be obtained according to Jespersen & ​Østergaard [11] and Mouridsen et al. [12], here referred to as apparent OEF (AOEF):

(6) $$AOEF=\int_{0}^{\infty }h\left(\tau \right)Q\left(\tau \right)d\tau ,$$

where h(τ) is the capillary transit time distribution and Q(τ) is oxygen extraction along a single capillary with transit time *τ*. The AOEF is a sum of the oxygen extraction in a single capillary over all transit times, weighted by the distribution of transit times over all capillaries. Determining the dependence of the single-capillary oxygen extraction Q on transit time τ requires a model, and here we follow the approach presented by Jespersen & ​Østergaard [11], where the capillary is treated as a three-compartment system comprising hemoglobin, oxygen in plasma and oxygen in tissue. The transport of oxygen across the capillary membrane is modelled as an exchange process described by a fixed rate constant *k*. Variation of oxygen concentration (C) along the capillary with a transit time τ is described by the differential equation in Eq. (7):

(7) $$\frac{dC}{dx}=-k\tau \left(\alpha_{H}P_{50}{\left(\frac{C}{B-C}\right)}^{1/h}-C_{tissue}\right)$$

where x is the normalized distance along the capillary, $\alpha_{H}=3.1\cdot 10^{-5}$ (mm Hg)^−1^ (Henry's constant), $P_{50}=26$ mm Hg (oxygen pressure required for half saturation), B=0.1943 ml/ml (maximum amount of oxygen bound to hemoglobin), h=2.8 (Hill coefficient) and $C_{tissue}=25$ mm Hg (tissue oxygen concentration) [11, 12]. From the solution of Eq. (7) above, Q is computed according to Q = 1−C(1)/C(0) as a function of *kτ*, enabling estimation of AOEF.

#### 2.1.3 Subjects and measurements

All measurements and associated postprocessing were approved by the local ethics committee (The Regional Ethical Review Board in Lund), and written informed consent was obtained from each subject. In light of ethically motivated restraints related to repeated injections of a gadolinium contrast agent in healthy volunteers, the current study relies on an independent re-analysis of image data acquired according to a previously reported data collection protocol [24, 25]. The current study is based on a separate scientific hypothesis and employs post-processing approaches and analyses that were not available at the time of the previous publications. Twenty healthy volunteers (10 females and 10 males in two age groups of equal size, 25–34 years and 51–84 years) were examined according to a test-retest protocol, with a time interval of 7–20 days between the two investigations (below referred to as visit 1 and visit 2). The volunteers underwent a neurological physical examination, including basic tests to verify their cognitive ability. The medical history of the volunteers was reviewed to exclude the risk that any medication could have altered their blood flow. Morphological images acquired before and after CA administration were evaluated to rule out the presence of any pathology.

Measurements were performed using a 3 T MRI unit (Philips Achieva, Philips Healthcare, Best, The Netherlands), and the *Smart-Exam* feature [26] was used for the planning of slice orientation and field of view (FOV). Gadolinium contrast agent (0.1 mmol/kg body weight, 5 ml/s, Dotarem, Guerbet, Paris, France) was injected, followed by a 20 ml saline flush at the same injection rate. DSC-MRI was carried out using single-shot GRE echo-planar imaging (EPI) with temporal resolution 1.24 s, FOV 220 × 220 mm^2^, image matrix 128 × 128, slice thickness 5 mm, 20 slices, flip angle 60°, TE 29 ms, and SENSE factor 2.5. The DSC-MRI experiment was preceded by a pre-bolus administration of a low dose of contrast agent, not further considered in the present study. The phase maps used for QSM-based OEF estimation were acquired using 3D GRE imaging with flow compensation. Magnitude and phase images were collected for 50 axial slices orthogonal to the external magnetic field, with spatial resolution 0.98 × 0.98 × 1.15 ${mm}^{3}$, field of view (FOV) = 220 × 220 ${mm}^{2}$, repetition time (TR) = 45 ms, TE = 20 ms, flip angle (FA) = 20°, bandwidth = 218 Hz/pixel.

### 2.2 Image processing and data analysis

#### 2.2.1 QSM-based OEF

QSM maps were reconstructed using the morphology-enabled dipole inversion (MEDI) toolbox (Cornell University, https://pre.weill.cornell.edu/mri/pages/qsm.html) [17, 27, 28, 29]. Spatial phase unwrapping was performed using a region growing algorithm [30] followed by background field removal using a projection onto dipole field (PDF) method [31]. The regularization parameter λ was set to 800. Voxels representing venous blood in the superior sagittal sinus (SSS) were selected using a previously published procedure [32]. In brief, a slab of interest (SOI) consisting of five consecutive slices was first identified manually in the QSM volume. In each SOI, smaller volumes of interest (VOIs), including the vessel of interest, were selected. The SSS was then outlined by segmentation of the VOI venous voxels using a venous mask, defining SSS values as all susceptibility values larger than the mean + 2 standard deviations (mean + 2SDs) of the corresponding SOI values. Finally, the values of SSS voxels assumed not to be affected by partial volume effects (PVEs) were obtained by averaging of the 20% highest values within the SSS. In the Δχ calculation, according to Eq. (2), the venous blood susceptibility was represented by this mean PVE-free SSS value, while the susceptibility value of the surrounding tissue was given by the mean value of the SOI, after exclusion of SSS voxels.

#### 2.2.2 MTT and AOEF obtained from DSC-MRI data

Tissue residue functions R(t) were retrieved according to Eq. (4) by pixelwise deconvolution of the tissue CA concentration curves with an AIF. Subject-specific global AIFs were measured in middle cerebral artery branches in the Sylvian fissure region. The employed deconvolution algorithm was based on Bézier curves including a compensation for time delays between the AIF and the tissue concentration curve [16]. The Bézier deconvolution approach was designed to return strictly decreasing residue curves, i.e., showing non-negative values without oscillations. MTT and AOEF were calculated according to Eq. 5 and Eq. 6, respectively. With regard to the rate constant *k*, one DSC-MRI dataset, originating from the volunteer that showed median QSM-based OEF level, was selected as a reference, and, similarly to previous approaches [12], *k* was set to return an oxygen extraction capacity value of 30% in a local white matter region in this dataset. This procedure yielded *k* = 25 s^−1^, and, in order to be able to compare AOEF estimates among subjects, this value of *k* was used in the analysis of all DSC-MRI datasets in this study. In our implementation, no attempts were made to interpret the AOEF values as absolute levels of oxygen extraction, i.e., the AOEF parameter should be understood as an OEF-related index in arbitrary units. In order to match the whole-brain OEF estimates obtained from QSM data, MTT and AOEF values were calculated as the mean value of all non-zero pixel values in the brain volume.

#### 2.2.3 Statistics

Repeatability was assessed by comparing results from visit 1 and visit 2 in Bland-Altman plots [33] and, for QSM-based OEF, by calculating intra-class correlation coefficients ICC(2,1) [two-way, absolute agreement, same rater, single measurement] and ICC(3,1) [two-way, consistency, same rater, single measurement] (MedCalc Software Ltd, Ostend, Belgium, version 20.109). The variability between subjects was evaluated by the coefficient of variation (CoV). The association between parameters was assessed by linear regression and correlation analysis (significance level α = 0.05). The assumptions of linear regression were assessed by testing for a linear relationship, testing for normal distribution of residuals and by visual inspection of residuals with respect to independence and homoscedasticity (see Supplementary Material 1).

## 3 Results

### 3.1 Assumptions of linear regression

The results of testing the assumptions of linear regression, for all datasets that were analyzed, are provided in Supplementary Material 1.

### 3.2 Absolute values

The OEF, MTT and AOEF results are summarized in Table 1, including population mean values, standard deviations (SDs) and the corresponding coefficients of variation (CoVs) between subjects. From the previous study by Knutsson et al. [24], a whole-brain CBF estimate is available for the investigated group of volunteers, with a group mean value of 43.6 ml/min/100 g. Applying this CBF value, in combination with the overall population mean QSM-based OEF value of 40.4 % from the current results, implies a population mean CMRO_2_ of 3.36 ml O_2_/min/100 g.

**Table 1:** Summary of OEF, MTT and AOEF results.

	OEF [%] Visit 1	OEF [%] Visit 2	MTT [s] Visit 1	MTT [s] Visit 2	AOEF [a.u.] Visit 1	AOEF [a.u.] Visit 2
Mean	39.3	41.4	3.20	3.25	0.207	0.203
SD	4.72	5.44	0.31	0.58	0.016	0.027
CoV	0.120	0.131	0.096	0.178	0.079	0.131

SD – standard deviation.

CoV – coefficient of variation between subjects.

### 3.3 Repeatability

**Figure 1:**
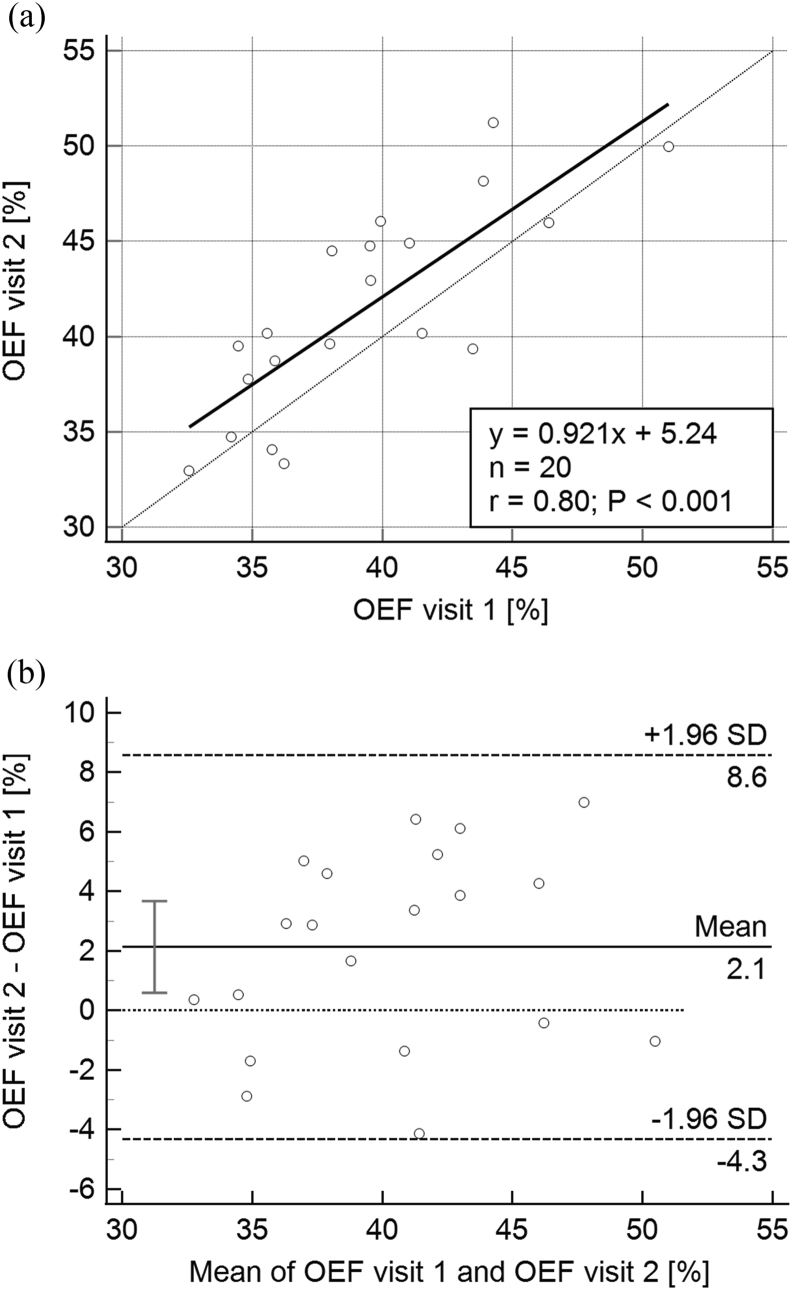
Test-retest results for QSM-based OEF estimates in healthy volunteers. (a) Scatter plot showing OEF from visit 2 versus OEF from visit 1. The solid line is the result of a linear regression analysis, and the dotted line is the line of identity. (b) Bland-Altman plot showing the OEF difference between visit 2 and visit 1 as a function of the mean OEF estimate from visit 1 and visit 2.

**Figure 2:**
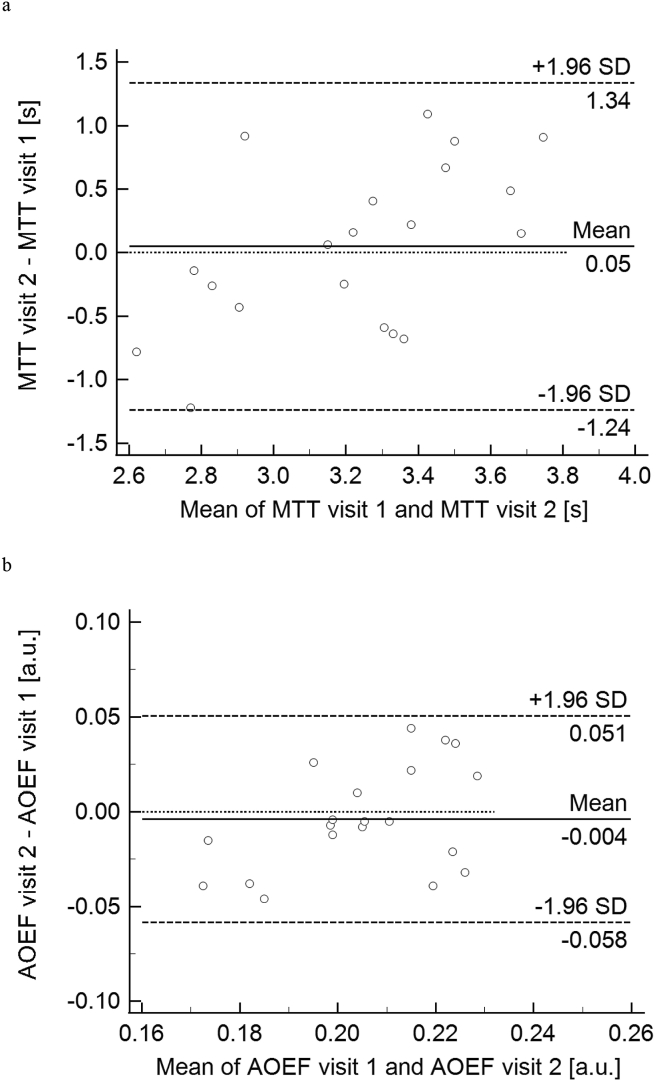
Bland-Altman plots of DSC-MRI-based (a) MTT and (b) AOEF estimates from visit 1 and visit 2, showing the difference between visit 2 and visit 1 as a function of the mean value from visit 1 and visit 2.

For OEF, a test-retest scatter plot (i.e., OEF visit 2 versus OEF visit 1) is displayed in Figure 1a and the corresponding Bland-Altman plot is shown in Figure 1b. The ICCs for the OEF test-retest data were ICC(2,1) = 0.73 and ICC(3,1) = 0.79. The repeatability features of MTT and AOEF are illustrated in the Bland-Altman plots in Figures 2a and 2b, respectively. Neither OEF, MTT nor AOEF test-retest data exhibited any outliers, according to the Bland-Altman analyses.

### 3.4 OEF and AOEF age dependences

**Figure 3:**
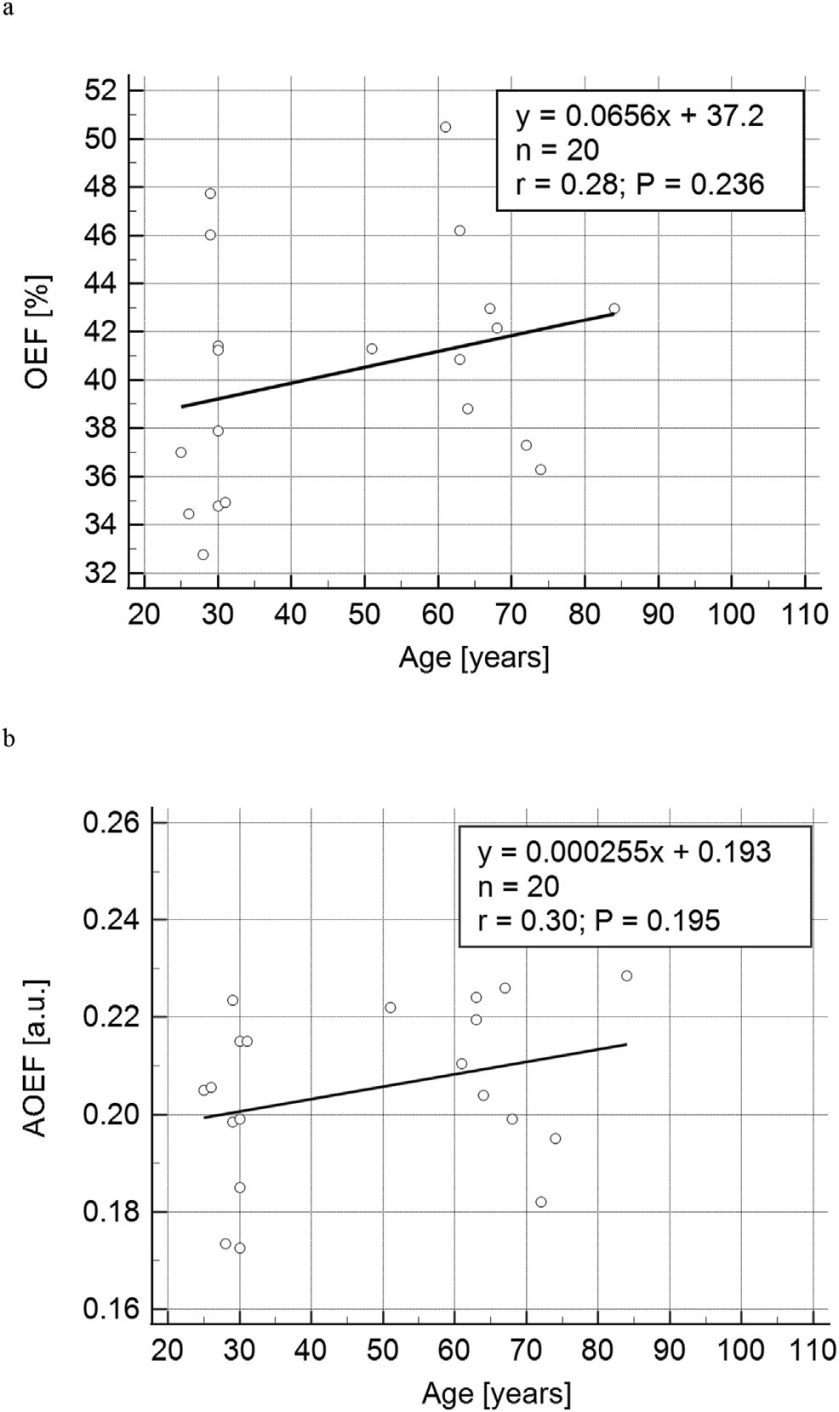
(a) QSM-based OEF (mean value of visit 1 and visit 2) as a function of age. The solid line is a trendline resulting from a linear regression analysis. (b) AOEF (mean value of visit 1 and visit 2) as a function of age. The solid line is a trendline resulting from a linear regression analysis.

The healthy volunteers were recruited to represent a large age interval, and Figures 3a and 3b show OEF and AOEF (mean values of visit 1 and visit 2), respectively, as a function of age. In Figures 3a and 3b, trendlines have been added for completeness, although the hypothesis that there is no linear relationship could not be rejected (see Supplementary Material 1). For OEF, the linear regression analysis implied an increase of approximately 0.066 percentage points/year.

### 3.5 Comparison between OEF and DSC-MRI parameters (MTT and AOEF)

**Figure 4:**
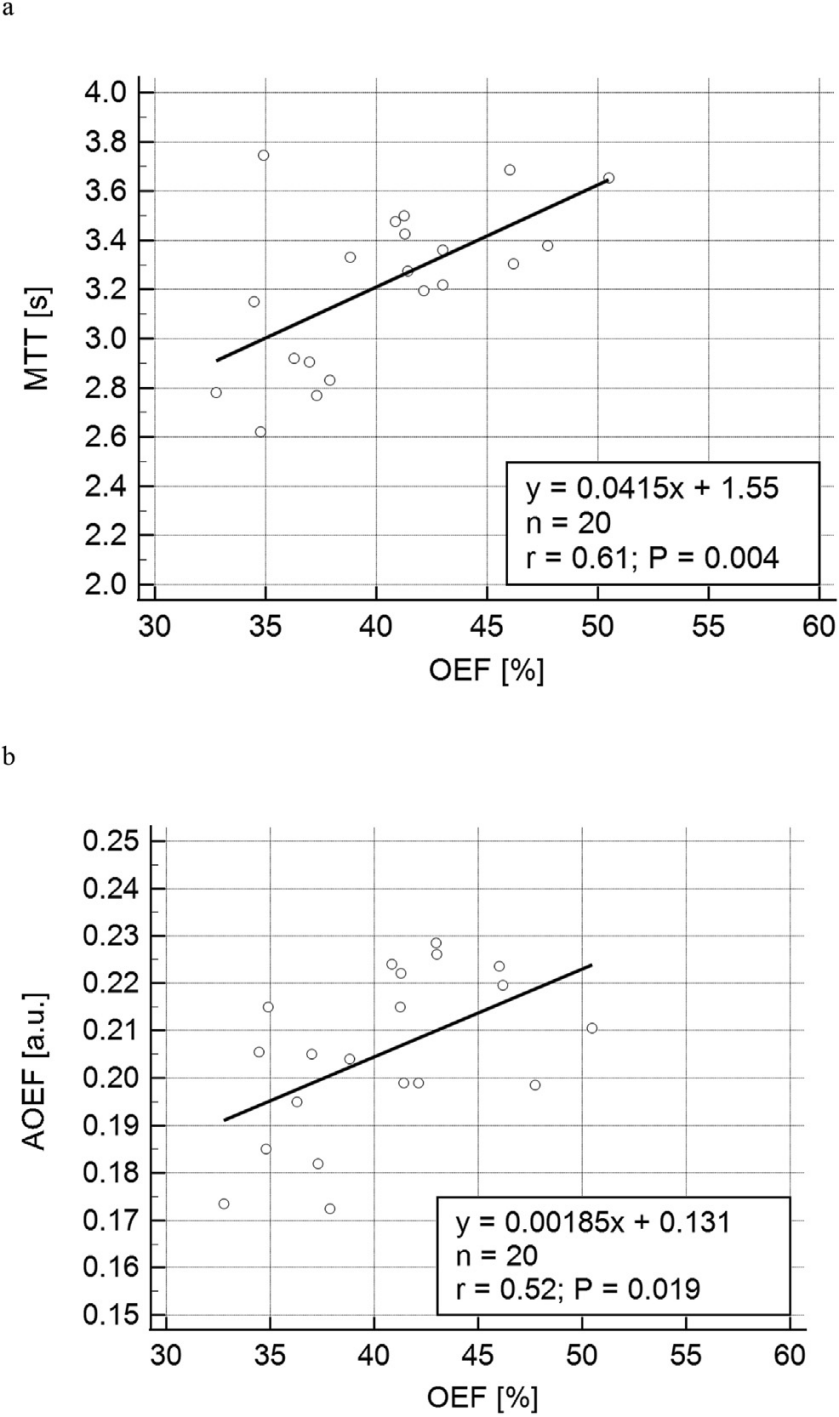
DSC-MRI-based estimates as a function of QSM-based OEF estimates (mean values of visit 1 and visit 2). Solid lines show the results of linear regression analyses. (a) MTT as a function of OEF. (b) AOEF as a function of OEF.

In the comparisons between OEF and MTT and between OEF and AOEF, the mean value of visit 1 and visit 2 is used to represent the respective parameter for each individual. The MTT-versus-OEF and AOEF-versus-OEF relationships are shown in Figures 4a and 4b, respectively, together with the results of a linear regression and correlation analysis. The association between MTT and OEF showed a correlation coefficient of r = 0.61 (p = 0.004) while the AOEF-versus-OEF relationship corresponded to r = 0.52 (p = 0.019).

### 3.6 Numerical data

Numerical data are available in Supplementary Material 2.

## 4 Discussion

The present study concerns evaluation of QSM-based OEF data as well as initial investigation of the potential usefulness of a DSC-MRI-based OEF index based on the tissue residue function retrieved by deconvolution. Data were acquired using equipment and imaging protocols that should be readily available in a normal clinical environment.

QSM reconstruction is a delicate mathematical procedure, and quantitative results are influenced by a number of factors, such as quality of phase data, phase data preprocessing, type of algorithm for inversion, regulariza& ​Østergaardtion settings, noise, brain mask extraction, etc. Hence, evaluation of QSM-based quantitative parameters are indeed warranted, and the general idea was to conduct the QSM reconstruction procedure and OEF analysis in a fashion that would be feasible to apply in a routine workflow.

Regarding the absolute level, QSM-based OEF values were in good agreement with literature data. The mean value of 40.4% and the full range 33–50% are quite consistent with reference levels from textbooks and other scientific studies. For example, ^15^O positron emission tomography (PET) OEF measurements have resulted in 39 ± 6 % [34] and 43 ± 6 % [35]. The full range (min-max) of OEF values reported by Hattori et al. was 30–51 % [34]. In this context, is should be noted that there has been some debate with regard to the correct value of Δχ_do_. Previously published values include Δχ_do_ = 0.18⋅4π ppm [1], Δχ_do_ = 0.2⋅4π ppm [36, 37] and Δχ_do_ = 0.27⋅4π ppm [38, 39]. In the present study, the result of the most recent investigation, focusing particularly on in vivo conditions, was employed, i.e., Δχ_do_ = 0.193⋅4π ppm [20]. Furthermore, the hematocrit level can be influenced by several factors, and the use of a constant value for all subjects is not optimal. In a crude attempt to take the gender differences into account, the values Hct = 0.42 for males and Hct = 0.40 for females were applied in this study, as suggested by Peng et al. [40].

The OEF repeatability, described by the ICC(2,1) = 0.73, was good and similar to other hemodynamic parameters evaluated in the same group of volunteers. For example, for DSC-MRI-based whole-brain cerebral blood volume (CBV) and CBF estimates, ICC values of 0.64–0.85 were reported [24]. The reported increase in OEF with age corresponding to 0.066 percentage points/year (Figure 3a) is clearly not statistically significant, and should not be overinterpreted. However, the current observation is, at least, not in contradiction to previously observed patterns of age dependence. For comparison, the comprehensive PET study by Leenders et al. reported an OEF increase of 0.13 percentage points/year in healthy volunteers [41].

The previously reported CBF estimate [24] for the investigated group of volunteers enabled an assessment of the cerebral metabolic rate of oxygen as well, serving as an additional indication of the methodological quality. The observed population mean CMRO_2_ of 3.36 ml O_2_/100 g/min is in excellent agreement with textbook values of the normal CMRO_2_ level, provided by Clarke & Sokoloff (approximately 3.5 ml O_2_/min/100 g in young adults) [42] and by Seubert & Mahla (range 3.0–3.5 ml O_2_/min/100 g) [43].

The MTT value of 3.2 s was, in absolute terms, somewhat short, but still comparable to other DSC-MRI studies [44]. Also, in some cases, previous DSC-MRI investigations may have arrived at overestimated MTT (=CBV/CBF), considering that conventional deconvolution algorithms have tended to underestimate CBF [16]. Bézier deconvolution is also available with delay compensation in combination with a vascular transfer function (VTF) for modelling of dispersion [16]. For the present data, the version including VTF resulted in comparable (even slightly better) correlation with QSM-based OEF (r = 0.67, p = 0.001 for MTT and r = 0.52, p = 0.018 for AOEF), but unrealistically short MTT values in absolute terms (population mean MTT <1.5 s) (data not shown). This MTT underestimation was predicted by simulation of cases where a VTF was applied to situations with low or absent arterial dispersion (as can be expected in healthy subjects) [16]. With regard to the relationships between DSC-MRI parameters and OEF, it should be remembered that correlation coefficients must, in general, be interpreted with caution. For example, the OEF variability between individuals in a healthy population is limited, and the correlation coefficient tends, in general terms, to become lower when the inter-subject variability is small. A positive correlation between OEF and MTT has been predicted by cerebral microvasculature modelling [45], and experimentally observed, for example, in a DSC-MRI and PET study of patients with chronic occlusive cerebrovascular disease [46] and in a PET study with acetazolamide test [47]. In the present study, it is particularly interesting that we, using Bézier deconvolution, were able to see a moderate-to-good significant correlation also within the limited range of healthy volunteers.

The AOEF parameter is considerably less explored and the exact nature of the information that it provides remains to be established. Hence, it is probably fair to state that the observed degree of association between OEF and AOEF was not below expectations. In this context, it should be pointed out that previous studies have used a large range of different rate constant (*k*) values [11, 12, 48]. This indicates that the exact choice of *k*, on group level, is not crucial for obtaining reasonable AOEF estimates in relative terms. However, it also suggests that the numerical setting of the *k* value is not entirely straightforward, and it cannot be completely ruled out that a further optimized rate constant *k* in this study might have resulted in a slightly altered correlation with OEF.

In pathophysiological situations, the residue function shape can become substantially altered compared with the normal case [49, 50]; one might speculate that the AOEF metric would provide better sensitivity, compared with MTT, under such conditions considering the fact that two residue curves with markedly different shapes can, in principle, show the same MTT. The AOEF-vs-OEF coefficient of correlation was moderate, but still significant (p < 0.02), which is reasonably encouraging under the circumstances. Considering that assessment of oxygen metabolism requires combined information about CBF and OEF (*cf.* Eq. 3), it would certainly be advantageous to be able to extract data related to both these physiological parameters from the same image dataset. An alternative approach, likely to be more robust at present, would be to obtain OEF by, for example, MRI susceptometry or QSM measurements and CBF from pseudo-continuous arterial spin labelling (ASL) [51]. A general drawback with standard ASL implementations is that they do not provide estimates of conventional CBV or MTT. However, the use of multiple post-label delays allows for the assessment of arterial cerebral blood volume (aCBV) and arterial arrival time (AAT) which may provide comparable information [52, 53].

As implied above, the phase data for QSM reconstruction were acquired with a slightly dated protocol, and this should be considered in the interpretation of our results. However, a recent 7 T study with an up-to-date protocol resulted in a very similar group mean OEF value of 42 % in healthy volunteers (when applying Δχ_do_ = 0.193⋅4π ppm and Hct = 0.40 for women and Hct = 0.42 for men), based on SSS QSM data and the same procedure of analysis as in the present study [32]. The DSC-MRI data acquisition in the present study was not regarded to be suboptimal in any way, and the preceding pre-bolus injection of a small dose of CA is not expected to negatively influence the main DSC-MRI experiment. For example, Jin et al. demonstrated, in a control experiment, that CBV estimates in normal (non-leaking) tissue were consistent between two sequential GRE-EPI DSC-MRI experiments (i.e. with repeated injections of Gd CA) [54], and, generally, DSC-MRI studies of brain tumours that are likely to show blood-brain barrier disruption frequently include administration of a small pre-bolus dose of CA to minimize T1 signal enhancement, caused by CA in the extravascular extracellular space [55]. Furthermore, the employed DSC-MRI deconvolution algorithm is novel and recently introduced by our group [16]. For ethical reasons, it would have been very difficult, or almost impossible, to undertake a similar test-retest contrast-agent injection scheme in healthy subjects today, and this was the main reason for employing a re-analysis of available datasets in this study.

Brain perfusion and related physiological parameters show variability or fluctuations over time and can be modified by a number of factors [56], and test-retest results may be influenced by such effects. However, systematic changes in resting-state OEF with age are not more than approximately 0.1 percentage points/year [41], so any such ageing effects should be negligible over approximately 1–3 weeks. With regard to short-term variability resulting from local brain activation, regional OEF is known to vary much less than CBF. Importantly, we investigated whole-brain estimates only, and neither whole-brain CBF nor whole-brain OEF is likely to vary significantly during local brain activation [57]. Furthermore, the aim of the present study was to assess parameters at rest, and the efficient autoregulation of the brain ensures that CBF at rest is fairly stable over time scales of days or weeks, as confirmed by previously reported ASL studies [58, 59].

In conclusion, QSM-based OEF showed a convincing absolute level and good test-retest results in terms of the ICC. Moderate-to-good correlations between DSC-MRI-based parameters (MTT and AOEF) and QSM-based OEF were observed. The results of the present study should be viewed upon as an indicator of the degree of robustness that can be achieved without applying extraordinary resources in terms of MRI equipment, imaging protocol, QSM preprocessing and reconstruction, and OEF analysis.

## Declarations

### Author contribution statement

Ronnie Wirestam: Conceived and designed the experiments; Performed the experiments; Analyzed and interpreted the data; Contributed reagents, materials, analysis tools or data; Wrote the paper.

Anna Lundberg; Arthur Chakwizira: Analyzed and interpreted the data; Contributed reagents, materials, analysis tools or data; Wrote the paper.

Danielle van Westen: Performed the experiments; Contributed reagents, materials, analysis tools or data.

Linda Knutsson: Performed the experiments; Contributed reagents, materials, analysis tools or data; Wrote the paper.

Emelie Lind: Performed the experiments; Analyzed and interpreted the data; Contributed reagents, materials, analysis tools or data; Wrote the paper.

### Funding statement

Professor Ronnie Wirestam was supported by Vetenskapsrådet [MH 2017-00995].

Emelie Lind was supported by Hjärnfonden [PS2021-0045].

### Data availability statement

Data will be made available on request.

### Declaration of interest’s statement

The authors declare the following conflict of interests: Under a license agreement between Philips and the Johns Hopkins University, Dr. Knutsson's spouse, Dr. van Zijl and the University are entitled to fees related to an imaging device used in the study discussed in this publication. Dr. van Zijl also is a paid lecturer for Philips. This arrangement has been reviewed and approved by the Johns Hopkins University in accordance with its conflict of interest policies.

### Additional information

Supplementary content related to this article has been published online at https://doi.org/10.1016/j.heliyon.2022.e12364.

## Appendix B Supplementary data

Supplementary Material 1 Supplementary Material 1

Supplementary Material 2.xlsx Supplementary Material 2.xlsx
