# Corrigendum to “Test-retest analysis of cerebral oxygen extraction estimates in healthy volunteers: Comparison of methods based on quantitative susceptibility mapping and dynamic susceptibility contrast magnetic resonance imaging” [Heliyon 8(12) (December 2022) e12364]

**DOI:** 10.1016/j.heliyon.2023.e21155

**Published:** 2023-10-21

**Authors:** Ronnie Wirestam, Anna Lundberg, Arthur Chakwizira, Danielle van Westen, Linda Knutsson, Emelie Lind

**Affiliations:** a Department of Medical Radiation Physics, Lund University, Lund, Sweden; b Department of Diagnostic Radiology, Lund University, Lund, Sweden; c Image and Function, Skåne University Hospital, Lund, Sweden; d F.M. Kirby Research Center for Functional Brain Imaging, Kennedy Krieger Institute, Baltimore, MD, United States; e Russell H. Morgan Department of Radiology and Radiological Science, Johns Hopkins University School of Medicine, Baltimore, MD, United States

In the original published version of this article, In the Materials and methods section, 2.1.2. Dynamic susceptibility contrast MRI under the equation 7 it reads: *C*_*tissue*_ = 25 mm Hg (tissue oxygen concentration) [11, 12].

The correct version can be found below: *C*_*tissue*_ = 25 mm Hg × 3.1⋅10^−5^ (mm Hg)^−1^ (tissue oxygen concentration in ml/ml) [11, 12].

The authors apologize for the error. The correction does not, in any way, affect the results presented in the article. Both the HTML and PDF versions of the article have been updated to correct the error.

