# Supplementary material for: Test-retest analysis of cerebral oxygen extraction estimates in healthy volunteers: comparison of methods based on quantitative susceptibility mapping and dynamic susceptibility contrast magnetic resonance imaging
Source: Heliyon. 2022 Dec 16;8(12):e12364. doi: 10.1016/j.heliyon.2022.e12364 (PMC9801129; doi:10.1016/j.heliyon.2022.e12364)
Supplement: Supplementary Material 1 [file mmc1.pdf]

Supplementary Material 1

Assessment of the assumptions of linear regression

The results displayed below were obtained using MedCalc® Statistical Software version 20.110 (MedCalc Software Ltd, Ostend, Belgium; https://www.medcalc.org; 2022)

Methods

Testing for linearity

Analysis of variance: The total variation in the dependent variable is divided into two components, one which can be attributed to the regression model (referred to as ”Regression”) and one which cannot be attributed to the regression model (referred to as ”Residual”). If the significance level for the F-test is less than 0.05, the hypothesis that there is no linear relationship can be rejected.

Testing for normal distribution of residuals

The Shapiro-Wilk test was applied. If P is higher than 0.05, data are assumed to have a normal distribution, and the conclusion '*Accept normality*' is shown. If P is less than 0.05, the conclusion '*Reject normality*' is shown.

Results

Figure 1a: Assessment of OEF Visit 2 versus OEF Visit 1 data

Testing for linearity: Analysis of variance

Source	DF	Sum of Squares	Mean Square
Regression	1	359.7898	359.7898
Residual	18	203.1559	11.2864

F-ratio	31.8781
Significance level	P<0.0001

Conclusion: The hypothesis that there is no linear relationship can be rejected.

Testing for normal distribution of residuals

Shapiro-Wilk test for normal distribution	W=0.9615 Accept normality (P=0.5747)

Conclusion: Data are assumed to have a normal distribution.

Residuals

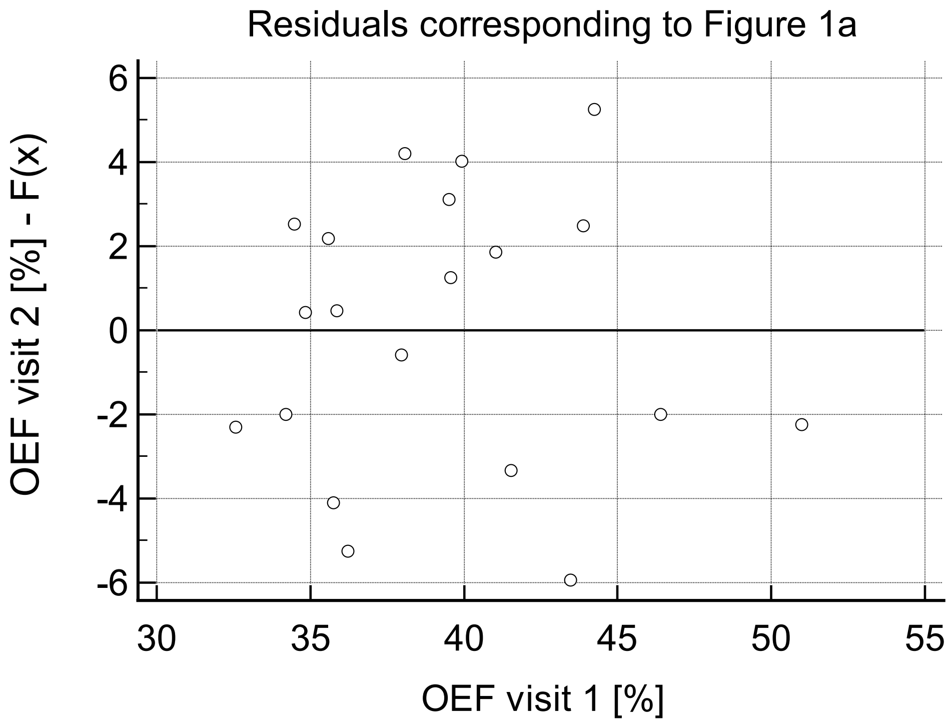

Conclusion: Visual inspection indicates that the assumptions of independence and homoscedasticity are not violated.

Figure 3a: Assessment of OEF versus age data

Testing for linearity: Analysis of variance

Source	DF	Sum of Squares	Mean Square
Regression	1	34.0197	34.0197
Residual	18	408.0390	22.6688

F-ratio	1.5007
Significance level	P=0.2363

Conclusion: The hypothesis that there is no linear relationship can not be rejected.

Testing for normal distribution of residuals

Shapiro-Wilk test for normal distribution	W=0.9350 Accept normality (P=0.1924)

Conclusion: Data are assumed to have a normal distribution.

Residuals

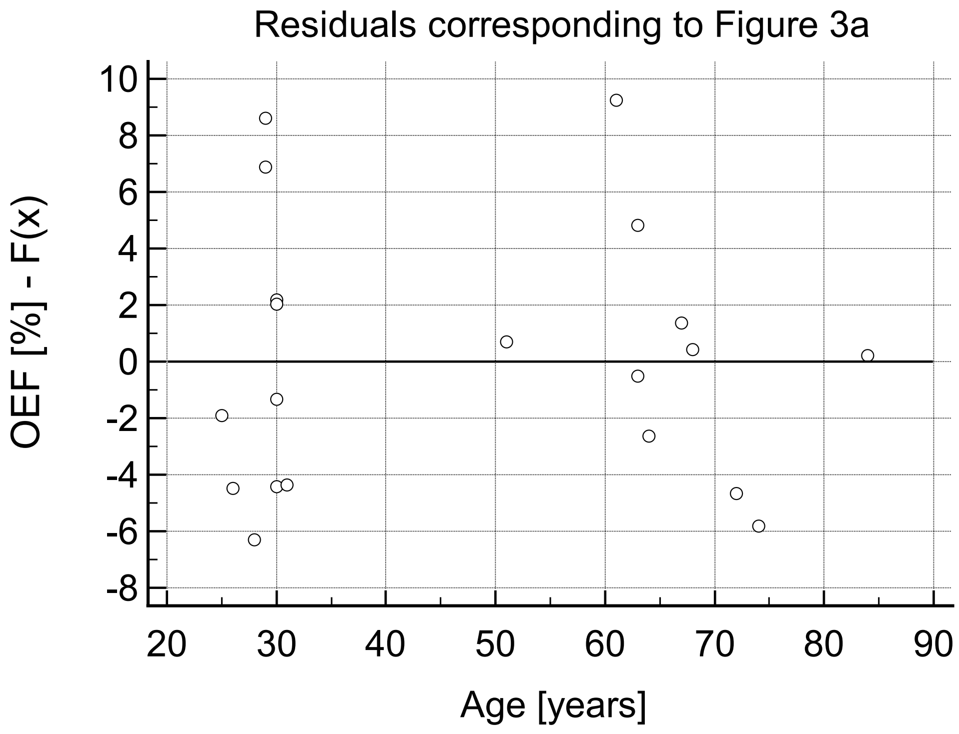

Conclusion: Visual inspection indicates that the assumptions of independence and homoscedasticity are not violated.

Figure 3b: Assessment of AOEF versus age data

Testing for linearity: Analysis of variance

Source	DF	Sum of Squares	Mean Square
Regression	1	0.0005141	0.0005141
Residual	18	0.005104	0.0002836

F-ratio	1.8128
Significance level	P=0.1949

Conclusion: The hypothesis that there is no linear relationship can not be rejected.

Testing for normal distribution of residuals

Shapiro-Wilk test for normal distribution	W=0.9117 Accept normality (P=0.0686)

Conclusion: Data are assumed to have a normal distribution.

Residuals

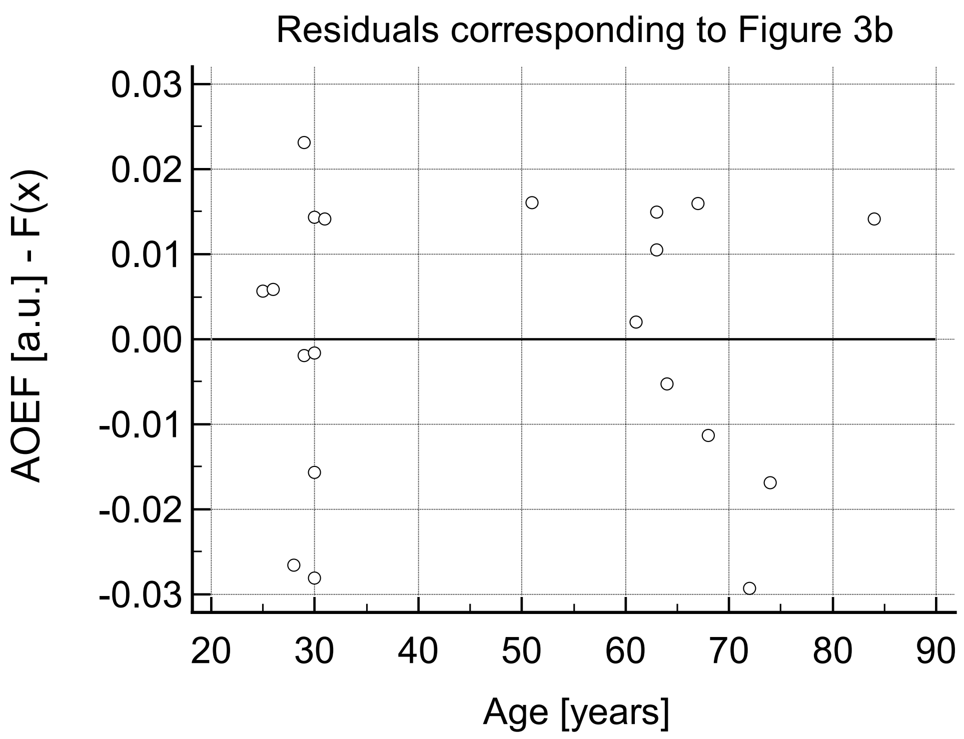

Conclusion: Visual inspection indicates that the assumptions of independence and homoscedasticity are not violated.

Figure 4a: Assessment of MTT versus OEF data

Testing for linearity: Analysis of variance

Source	DF	Sum of Squares	Mean Square
Regression	1	0.7620	0.7620
Residual	18	1.2743	0.07079

F-ratio	10.7638
Significance level	P=0.0042

Conclusion: The hypothesis that there is no linear relationship can be rejected.

Testing for normal distribution of residuals

Shapiro-Wilk test for normal distribution	W=0.9100 Accept normality (P=0.0637)

Conclusion: Data are assumed to have a normal distribution.

Residuals

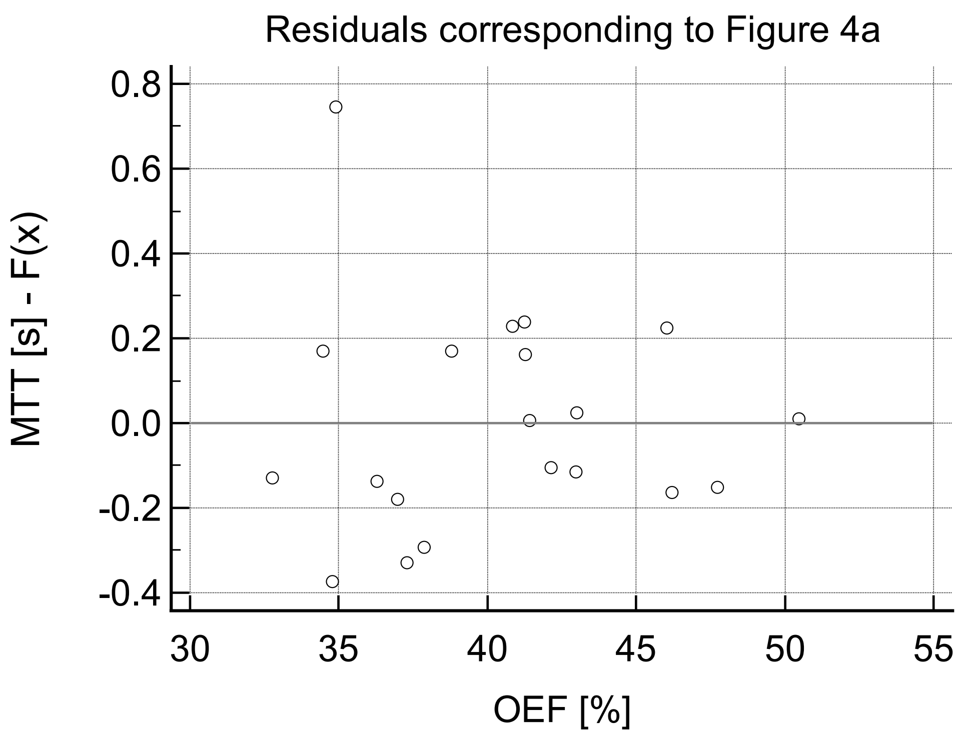

Conclusion: Visual inspection indicates that the assumptions of independence and homoscedasticity are not violated.

Figure 4b: Assessment of AOEF versus OEF data

Testing for linearity: Analysis of variance

Source	DF	Sum of Squares	Mean Square
Regression	1	0.001511	0.001511
Residual	18	0.004108	0.0002282

F-ratio	6.6210
Significance level	P=0.0191

Conclusion: The hypothesis that there is no linear relationship can be rejected.

Testing for normal distribution of residuals

Shapiro-Wilk test for Normal distribution	W=0.9423 Accept normality (P=0.2645)

Conclusion: Data are assumed to have a normal distribution.

Residuals

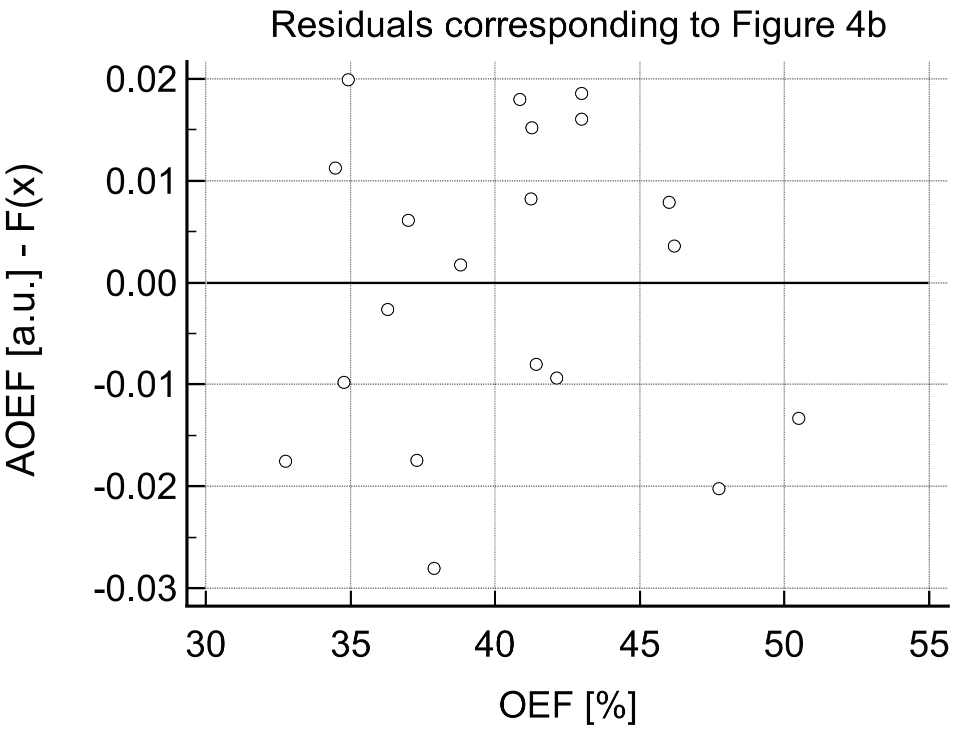

Conclusion: Visual inspection indicates that the assumptions of independence and homoscedasticity are not violated.
